# BET inhibitors synergize with sunitinib in melanoma through GDF15 suppression

**DOI:** 10.1038/s12276-023-00936-y

**Published:** 2023-02-01

**Authors:** Furong Zeng, Yayun Li, Yu Meng, Huiyan Sun, Yi He, Mingzhu Yin, Xiang Chen, Guangtong Deng

**Affiliations:** 1 Department of Dermatology, Hunan Engineering Research Center of Skin Health and Disease, Hunan Key Laboratory of Skin Cancer and Psoriasis, Xiangya Clinical Research Center for Cancer Immunotherapy, Xiangya Hospital, Central South University, Changsha, Hunan China; 2 Department of Oncology, Xiangya Hospital, Central South University, Changsha, Hunan China; 3 National Clinical Research Center for Geriatric Disorders, Xiangya Hospital, Central South University, Changsha, Hunan China; 4 National Engineering Research Center of Personalized Diagnostic and Therapeutic Technology, Changsha, Hunan China

**Keywords:** Melanoma, Translational research

## Abstract

Targeting bromodomain and extra-terminal domain (BET) proteins has shown a promising therapeutic effect on melanoma. The development of strategies to better kill melanoma cells with BET inhibitor treatment may provide new clinical applications. Here, we used a drug synergy screening approach to combine JQ1 with 240 antitumor drugs from the Food and Drug Administration (FDA)-approved drug library and found that sunitinib synergizes with BET inhibitors in melanoma cells. We further demonstrated that BET inhibitors synergize with sunitinib in melanoma by inducing apoptosis and cell cycle arrest. Mechanistically, BET inhibitors sensitize melanoma cells to sunitinib by inhibiting GDF15 expression. Strikingly, GDF15 is transcriptionally regulated directly by BRD4 or indirectly by the BRD4/IL6/STAT3 axis. Xenograft assays revealed that the combination of BET inhibitors with sunitinib causes melanoma suppression in vivo. Altogether, these findings suggest that BET inhibitor-mediated GDF15 inhibition plays a critical role in enhancing sunitinib sensitivity in melanoma, indicating that BET inhibitors synergize with sunitinib in melanoma.

## Cancer: a drug combination for combating melanoma

Drugs that target proteins involved in issuing stop and start commands to cancer-related genes help to sensitize melanoma cells to a widely used anti-cancer therapy, leading to tumor shrinkage in mice. Furong Zeng from Central South University in Changsha, China, and colleagues screened 240 approved anti-cancer agents in search of molecules that work synergistically with a BET inhibitor, an inhibitor of these proteins, to induce the death of melanoma cells. They found that a protein targeted by BET inhibitors (JQ1 and NHWD-870) regulates a growth factor protein, whose altered expression then helps to sensitize melanoma cells to sunitinib. Sunitinib is approved to treat several types of cancer and works by targeting many different kinds of kinase enzymes. The combination of BET inhibitors and sunitinib showed synergistic antitumor effects in a mouse model of melanoma.

## Introduction

The introduction of targeted therapies and immunotherapy has improved survival outcomes in the majority of melanoma patients^1^. However, there are still a large number of patients whose outcomes are dismal because of dose-limiting toxicity and innate or acquired resistance^2^. Multiple causes of resistance to targeted therapies have been clarified, including reactivation of the MAPK pathway and activation of alternate receptor kinase pathways^3,4^. Similarly, resistance to immunotherapy can occur through the Janus kinase (JAK1/2) cytokine signaling pathway, with reduced PD-1 ligand (PD-L1) expression or decreased beta2-microglobulin expression causing the loss of major histocompatibility complex class 1 expression^5–7^. Therefore, it is still of great significance to explore new individualized targeted therapies for melanoma.

Targeting bromodomain and extra-terminal domain (BET) proteins has become an attractive antitumor strategy due to their abnormal expression and promotion of melanoma carcinogenesis^8^. Our team previously developed a novel oral BET inhibitor, NHWD-870, which could suppress cancer cell-macrophage interactions through the BRD4/HIF1a/CSF1 axis^9^. We further demonstrated that BET inhibitors could inhibit melanoma progression through the noncanonical NF-κB/SPP1 pathway^10^. In addition, many excellent studies have shown that BET inhibitors can modulate sensitivity to other drugs. For example, Yang et al. demonstrated that BET inhibitors sensitized homologous recombination-proficient cancers to poly(adenosine diphosphate–ribose) polymerase (PARP) inhibitors^11^. Kanojia et al. showed that BET inhibitors induced βIII-tubulin expression and sensitized metastatic breast cancer in the brain to vinorelbine^12^. Thus, the development of strategies to better kill melanoma cells with BET inhibitor treatment may provide new clinical applications.

Combination drug therapy is an effective strategy to improve drug efficacy in melanoma. Researchers found that BET inhibitors synergized with RAF/BRAF/MEK inhibitors in melanoma^13–18^. Moreover, cotargeting BET and CDK9 had synergistic effects against melanoma cells in vitro and in vivo^19^. However, these combination strategies are far from clinical use, highlighting the importance of screening the Food and Drug Administration (FDA)-approved drug library to identify drugs that synergize with BET inhibitors in melanoma cells.

Sunitinib, a novel oral multitargeted tyrosine kinase inhibitor approved by the FDA in 2006, is used to treat patients with clear cell renal cell carcinoma or gastrointestinal stromal tumors^20^. Our team previously demonstrated that sunitinib had a therapeutic effect on melanoma that was further enhanced by propranolol^21^. However, whether BET inhibitors are involved in the regulation of sunitinib sensitivity is completely unknown. Here, we identified sunitinib among 240 FDA-approved antitumor drugs to synergize with BET inhibitors in melanoma cells. Mechanistically, BET inhibitors sensitize melanoma cells to sunitinib by inhibiting GDF15 expression. Strikingly, GDF15 is transcriptionally regulated directly by BRD4 or indirectly by the BRD4/IL6/STAT3 axis. Xenograft assays revealed a reduction in tumor volume with combined BET inhibitor and sunitinib treatment. Altogether, these findings suggest that BET inhibitor-mediated GDF15 inhibition plays a critical role in enhancing sunitinib sensitivity, indicating that BET inhibitors synergize with sunitinib in melanoma.

## Materials and methods

### Cell culture

A375, SK-MEL-28, and HEK293T cells were obtained from the American Type Culture Collection (ATCC, VA, USA). All cell lines were cultured in Dulbecco’s modified Eagle’s medium (Biological Industries) supplemented with 10% fetal bovine serum (Biological Industries) and 1% penicillin–streptomycin solution (Beyotime Biotechnology) in a humidified 37 °C incubator with 5% CO_2_.

### Reagents and antibodies

FDA-approved antitumor drugs were purchased from Selleck Chemicals (Houston, TX, USA). Antibodies specific for the following proteins were used: STAT3 (#9139, Cell Signaling Technology, MA, USA), phospho-Stat3 (Tyr705) (#9145, Cell Signaling Technology, MA, USA), phospho-Stat3 (Ser727) (#49081, Cell Signaling Technology, MA, USA), BRD2 (#ab139690, Abcam, Wales, UK), BRD3 (#ab50818, Abcam, Wales, UK), BRD4 (#ab128874, Abcam, Wales, UK), GDF15 (27455-1-AP, Proteintech, Wuhan, Hubei, China), Ki67 (#ab15580, Abcam, Wales, UK) and ACTIN (#sc-8432, Santa Cruz Biotechnology, TX, USA).

### Cell cycle and cell apoptosis

Cell cycle analysis was performed by flow cytometry using a cell cycle kit (Beyotime, C1052) as previously described^2^. The cell cycle distribution was assessed by FlowJo. Apoptosis was assessed with an Annexin V-AF647/PI kit (4 A Biotech, FXP023-050) by flow cytometry.

### Lentiviral transduction and RNA interference

Stable cell lines were generated as described previously^22^. Transfection with shRNA, siRNA, or cDNA was performed with TurboFect (Thermo Fisher Scientific, R0531) according to the manufacturer’s instructions. STAT3 shRNA was purchased from GeneChem. The wild-type and mutant STAT3 sequences were inserted in the pCDH-3xFLAG-GFP-puroR vector obtained from Youze Biotechnology, and the siRNA sequences were obtained from RiboBio (siSTAT3#1: GCAACAGATTGCCTGCATT; siSTAT3#2: CAACATGTCATTTGCTGAA.). The knockdown efficiency was quantified by real-time PCR and western blotting. The guide RNA sequences constituted a pool of two different sgRNA plasmids to target human GDF15, and the sgRNA sequences were as follows: GAAACTTGCGCGGCTCGCCT, TTCGAACACCGACCTCGTCC.

### RNA extraction and real-time PCR

Total RNA was extracted using MagZol (Magen, R4801). cDNA was generated using a HiScript Q RT SuperMix kit (Vazyme, R223-01). Real-time PCR was performed with SYBR Green Master Mix (Bimake, B21703). GAPDH was used as an internal control. The following primers were used:

CDK1-F: GGAAACCAGGAAGCCTAGCATC;

CDK1-R: GGATGATTCAGTGCCATTTTGCC;

CDC6-F: GGAGATGTTCGCAAAGCACTGG;

CDC6-R: GGAATCAGAGGCTCAGAAGGTG;

IL6-F: AGACAGCCACTCACCTCTTCAG;

IL6-R: TTCTGCCAGTGCCTCTTTGCTG;

GDF15-F: CAACCAGAGCTGGGAAGATTCG;

GDF15-R: CCCGAGAGATACGCAGGTGCA;

STAT3-F: CTTTGAGACCGAGGTGTATCACC;

STAT3-R: GGTCAGCATGTTGTACCACAGG;

GAPDH-F: AATCCCATCACCATCTTCCA

GAPDH-R: GTCATCATATTTGGCAGGTT

### Western blotting

Cell lysates were prepared in NP-40 buffer (Beyotime, P0013F). Protein extracts were analyzed by western blotting according to the manufacturer’s protocol, as previously described^22^.

### Cell viability

Cell viability was measured using the Cell Counting Kit-8 (CCK-8) assay (Bimake, B34302) as previously described^2^. The combination index (CI) was calculated using CompuSyn software based on the Chou-Talalay method, and a CI of less than 1 indicated synergy.

### Chromatin immunoprecipitation (ChIP)-qPCR and sequencing

ChIP-qPCR and sequencing were performed as previously described^2^. Sonicated samples were immunoprecipitated with antibodies against STAT3 (Cell Signaling Technology, 9139 S) and BRD4 (Cell Signaling Technology, 13440). The primers used were as follows: GDF15-Chip-F, GGCAAGAACTCAGGACGGTG; GDF15-Chip-R, TCGTAGCGTTTCCGCAACT. IL6-Chip-F, GACATGCCAAAGTGCTGAGTC; IL6-Chip-R, ACTAGGGGGAAAAGTGCAGC.

### RNA-seq

A375 cells were treated with DMSO, 4 μM sunitinib, 1 μM JQ-1, or 4 μM sunitinib + 1 μM JQ-1 for 24 h. Total RNA was extracted with MagZol and used for RNA-seq analysis. Libraries were constructed and sequenced on a BGISEQ-500RS sequencer. Only genes with at least 1 read in each of the six samples and at least 50 reads in total among all samples were retained for subsequent analyses. Differentially expressed genes were defined as those with a | log2-fold-change | > 1 and *q* value < 0.05.

### Bioinformatics analysis

The gene expression profile datasets GSE78864, GSE122819 and GSE122821 were downloaded from the Gene Expression Omnibus (GEO) database. Genes were ranked according to the shrunken limma log2-fold changes, and the GSEA tool was used in ‘preranked’ mode with all default parameters. GSEA was performed using Java desktop software (http://software.broadinstitute.org/gsea/index.jsp). For our own RNA-seq data, GSVA and GSEA were performed using R software (3.6.3). The normalized counts were fit to a negative binomial GLM for differential expression analysis using edgeR.

### TUNEL assay

Apoptotic cells in tumor tissue were evaluated by a TUNEL assay (Beyotime Biotechnology, C1089) as previously described^2^. The slides were then counterstained with DAPI (Servicebio, Wuhan, China). Positively stained cells were examined by microscopy.

### ELISA for measurement of the IL6 concentration

Melanoma cells were seeded in 6-well plates and cultured overnight. After treatment, the supernatants were collected, and the IL6 concentration was measured using a human IL6 Valukine ELISA Kit (#VAL102, Novus, CO, USA) according to the manufacturer’s protocols.

### Animal study

All animal experiments were performed in accordance with protocols approved by the Ethical Review of Experimental Animals committee at Central South University. A375 cells (2 × 10^6^) were suspended in 100 μl of PBS and inoculated into nude mice (Shanghai SLAC). Tumor-bearing mice were randomly allocated into groups. When the tumor volume reached 50-100 mm^3^, the mice were treated with vehicle (corn oil + citrate buffer (0.1 mol/L, pH = 3.5), orally), NHWD-870 (in corn oil, 0.75 mg/kg, orally), sunitinib (in citrate buffer, 40 mg/kg, orally), or a combination of both drugs for two days, and treatment was then stopped for one day. This process was repeated for the duration of the treatment period. The tumor size was recorded every three days, and the volume was calculated as [(length × width × width) / 2].

### Statistical analyses

All the data are presented as the means ± SDs and were analyzed with GraphPad Prism 8. Two-tailed unpaired Student’s t test was employed for comparisons between two groups. ANOVA was performed for comparisons among multiple groups. Nonparametric tests were applied if the data were nonnormally distributed. A *P* value of <0.05 was considered statistically significant.

## Results

### Identification of synergy between sunitinib and BET inhibitors in melanoma

**Fig. 1:**
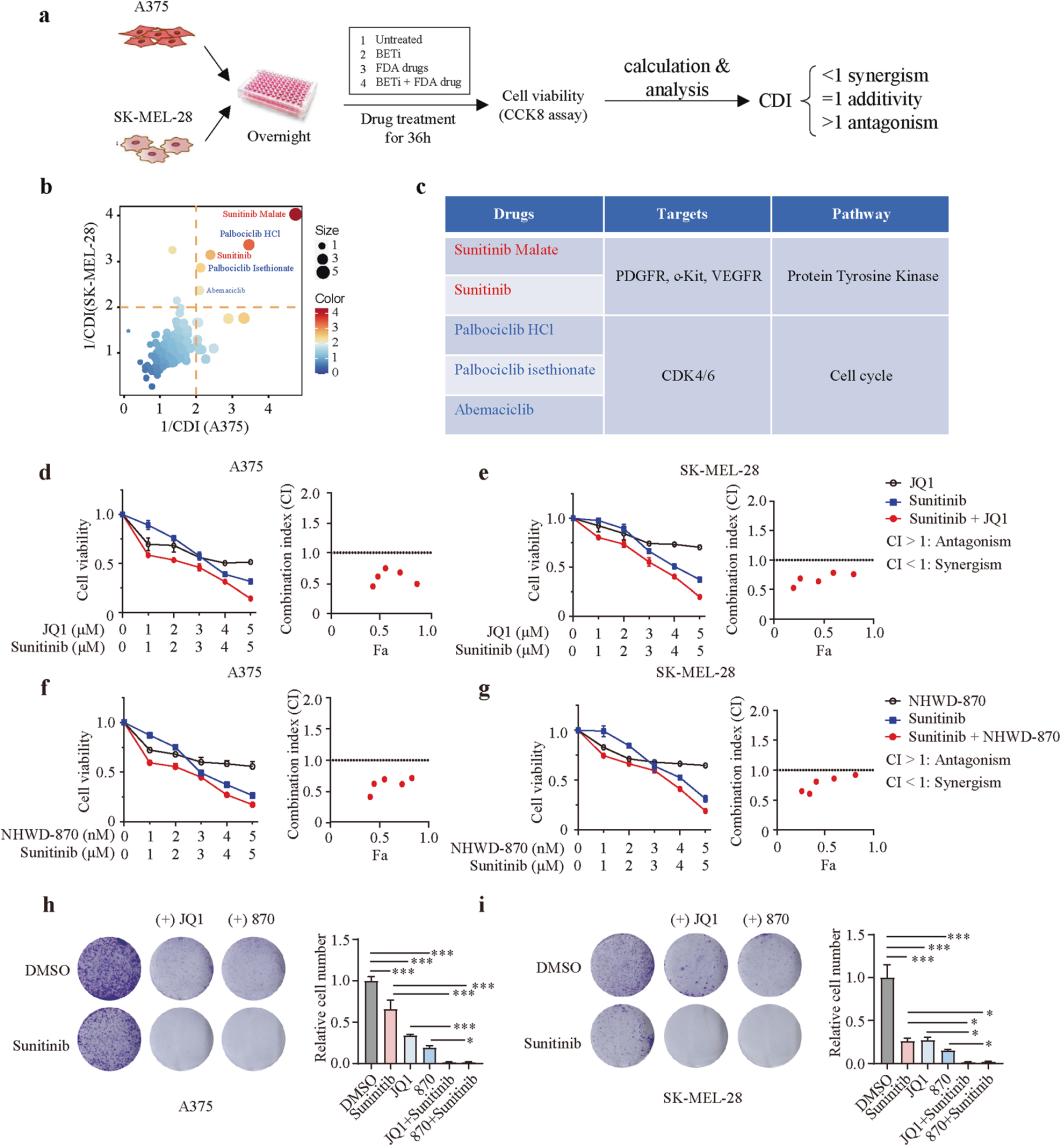
Identification of synergy between sunitinib and BET inhibitors in melanoma cells. **a** Schematic of the screening process for identifying clinically applicable drugs from the FDA-approved drug library that synergize with BET inhibitors in melanoma. **b** Summary scatter plot of CDI values in A375 and SK-MEL-28 cells. **c** Targets and targeted pathways of the drugs identified through the screen. **d**–**g** Dose-response curves of melanoma cells treated with sunitinib or JQ1/NHWD-870 either alone or in combination for 36 h (JQ1 and sunitinib at a fixed ratio of 1:1, NHWD-870 and sunitinib at a fixed ratio of 1:1000). Synergy was assessed by the Chou-Talalay combination index (CI) for sunitinib and BET inhibitors across the indicated cell lines. The *x*-axis on the CI plots shows the percentage of cells affected. **h**, **i** Colony formation assay of A375 and SK-MEL-28 cells after the indicated treatment. P values were calculated using one-way ANOVA in (**h**) and (**i**). *P < 0.05; ***P < 0.001.

To explore potential drugs from the FDA-approved drug library that synergize with BET inhibitors in melanoma, we performed a screen of 240 antitumor drugs combined with JQ1 using an in vitro drug combination assay (Fig. 1a). The coefficient of drug interaction (CDI) was used to assess the effect of the combination treatments^23^. Sunitinib was identified as one of the most promising drugs in both A375 and SK-MEL-28 cells, in addition to CDK4/6 inhibitors, which have been reported to synergize with BET inhibitors in cancer cells, thus supporting the validity of our screen (Fig. 1b, c). To further clarify whether JQ1 synergizes with sunitinib, we conducted a dose-response experiment with increasing drug concentrations in both A375 and SK-MEL-28 cells. The combination index (CI) was used to evaluate the interactions between BET inhibitors and sunitinib in CompuSyn software using the Chou-Talalay method. The CI values in both melanoma cell lines tended to be less than 1 and thus suggested a synergistic effect (Fig. 1d, e). Consistent with this finding, NHWD-870, another BET inhibitor we developed^9^, also showed strong synergistic effects with sunitinib in both A375 and SK-MEL-28 cells (Fig. 1f, g). In addition, to investigate the antiproliferative effect of cotreatment with BET inhibitors and sunitinib, a colony formation assay was performed, and the results showed that compared with sunitinib or BET inhibitors alone, combination treatment with sunitinib and BET inhibitors significantly suppressed melanoma cell proliferation (Fig. 1h, i). These findings suggested that BET inhibitors synergize with sunitinib in melanoma.

### Apoptosis and cell cycle arrest mediate the synergy induced by the combination treatment

**Fig. 2:**
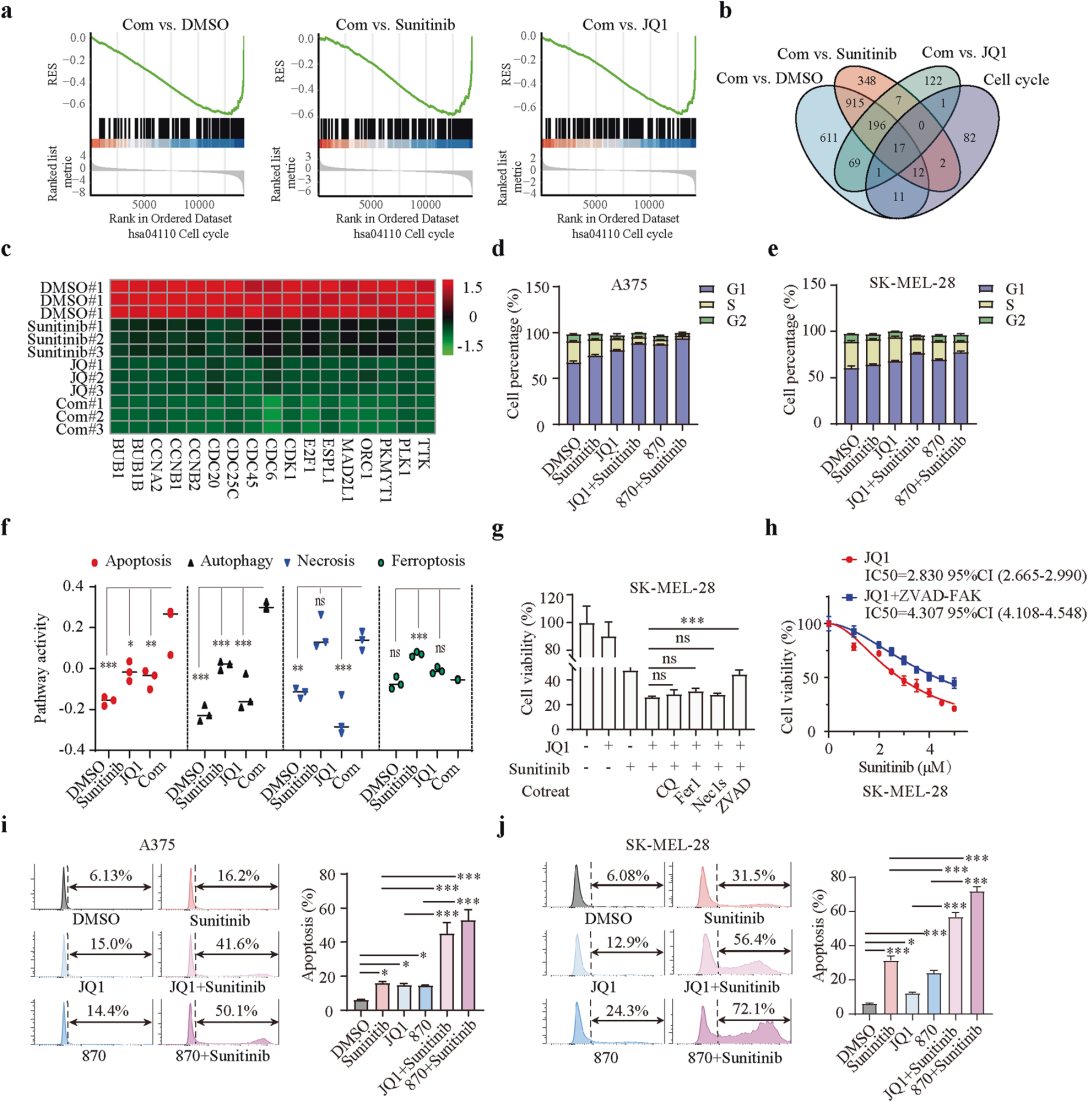
Apoptosis and cell cycle arrest mediate the synergy induced by the combination treatment. **a** Gene set enrichment analysis (GSEA) showing that cell cycle progression was significantly inhibited in the combination group compared with the DMSO, sunitinib, and JQ1 groups. **b** Venn diagram of the overlapping genes in the indicated groups. **c** Heatmap of the 17 genes identified by the Venn diagram that were associated with the cell cycle and inhibited by the combination treatment. **d**, **e** Cell cycle distribution of A375 (**d**) and SK-MEL-28 (**e**) cells after treatment with sunitinib (1 μM) or JQ1 (1 μM)/NHWD-870 (10 nM) either alone or in combination for 36 h. **f** Gene set variation analysis (GSVA) of apoptosis-, autophagy-, necrosis-, and ferroptosis-related pathways in the indicated groups. **g** SK-MEL-28 cells were treated with JQ1 (1 μM), sunitinib (1 μM), or a combination of both drugs with or without cell death inhibitors (CQ, 10 μM; Fer-1, 2 μM; necrostatin-1s, 10 μM; ZVAD-FMK, 5 μM) for 24 h, and cell viability was assessed. **h** Dose response of sunitinib-induced death of SK-MEL-28 cells treated with JQ1 in the absence or presence of ZVAD-FAK. **i**, **j** Apoptosis of A375 (**i**) and SK-MEL-28 (**j**) cells after treatment with sunitinib (1 μM) or JQ1 (1 μM)/NHWD-870 (10 nM) either alone or in combination for 36 h. *P* values were calculated using one-way ANOVA in (**f**, **g**, **i** and **j**). *P < 0.05; **P < 0.01; ***P < 0.001; ns nonsignificant.

To further clarify the underlying mechanism of the synergy induced by combination treatment with BET inhibitors and sunitinib, we performed RNA-seq analysis on the following groups of A375 melanoma cells treated for 24 h as indicated: control; JQ1, 1 µM; sunitinib, 4 µM; combination, JQ1, 1 µM and sunitinib, 4 µM. Gene set enrichment analysis (GSEA) was performed and showed that cell cycle progression was significantly inhibited in the combination group compared with the other groups (Fig. 2a). To determine which genes were suppressed in the cell cycle gene set, we drew a Venn diagram and heatmap and found that 17 genes associated with the cell cycle were dramatically inhibited by cotreatment with BET inhibitors and sunitinib (Fig. 2b, c). Among these genes, Cyclin Dependent Kinase 1 (CDK1) is best known for its function as a master regulator of the cell cycle^24^. Cell division cycle 6 (CDC6) is an essential regulator of DNA replication in eukaryotic cells^25^. The changes in the expression of these genes were confirmed by quantitative real-time PCR analysis (Supplementary Fig. 1a, b). We further evaluated cell cycle progression in both A375 and SK-MEL-28 cells using flow cytometry. We found that cotreatment with BET inhibitors and sunitinib significantly induced G1 arrest compared with sunitinib or BET inhibitors alone (Fig. 2d, e). We also used gene set variation analysis (GSVA) to explore the effects of the drug treatments on cell death-related pathways, including apoptosis, autophagy, necrosis and ferroptosis. We found that apoptosis and autophagy pathways were dramatically activated in the combination group compared with the other groups (Fig. 2f). Furthermore, the toxic effect of the combination therapy was partially negated by an inhibitor of apoptosis (Z-VAD-FMK) but not by inhibitors of ferroptosis (ferrostatin-1), necroptosis (Nec-1s), or autophagy (CQ) (Fig. 2g). Consistent with these results, Z-VAD-FMK still partially inhibit the death of A375 cells induced by cotreatment with JQ1 and different concentrations of sunitinib (Fig. 2h). Flow cytometry further demonstrated that treatment with BET inhibitors in combination with sunitinib resulted in a much higher proportion of apoptotic cells than sunitinib or BET inhibitors alone (Fig. 2i, j; Supplementary Fig. 1c, d), suggesting that the combination of BET inhibitors and sunitinib drives melanoma cell apoptosis. Altogether, these results indicated that cotreatment with BET inhibitors and sunitinib inhibits melanoma progression by significantly promoting cell cycle arrest and apoptosis.

### STAT3 regulates sunitinib sensitivity in melanoma cells through its phosphorylation

**Fig. 3:**
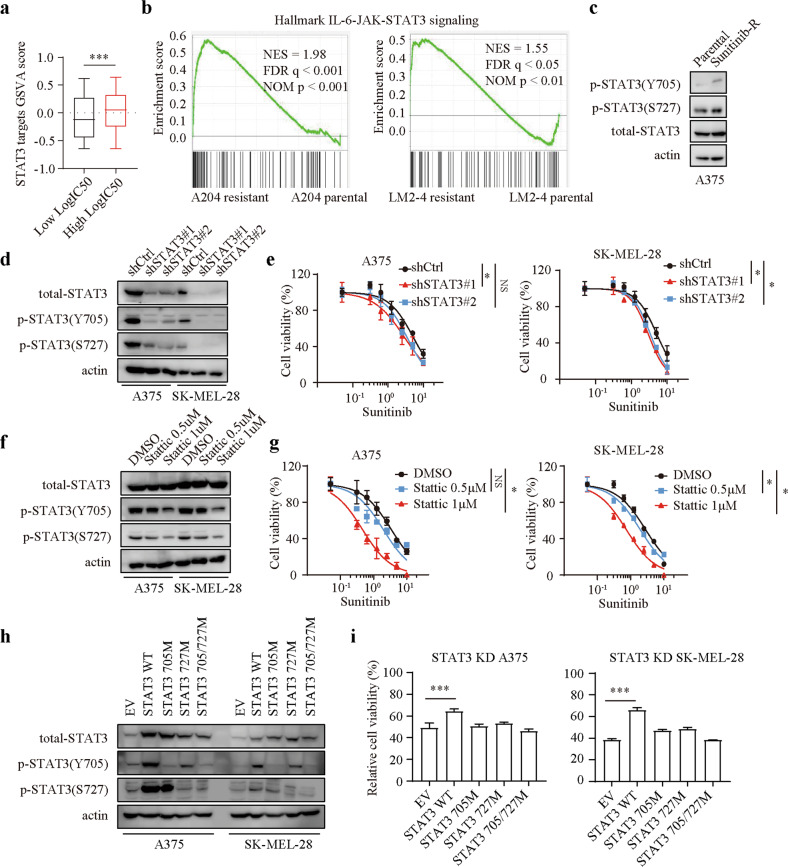
STAT3 regulates sunitinib sensitivity in melanoma cells through its phosphorylation. **a** GSVA scores of STAT3 targets in cells with a high IC50 of sunitinib or a low IC50 of sunitinib from the Cancer Therapeutics Response Portal dataset. **b** GSEA of the hallmark IL6/JAK/STAT3 signaling pathway in the indicated parental and sunitinib-resistant cells. **c** Western blot analysis of the indicated proteins in parental and sunitinib-resistant A375 cells. **d** Western blot analysis of the indicated proteins in shCtrl and shSTAT3 melanoma cells. **e** Dose response of sunitinib-induced death in shCtrl and shSTAT3 melanoma cells over a 24 h period. **f** Western blot analysis of the indicated proteins in A375 and SK-MEL-28 cells after treatment with DMSO, 0.5 μM stattic, or 1 μM stattic for 24 h. **g** Dose response of sunitinib-induced death in A375 and SK-MEL-28 cells in the presence of DMSO, 0.5 μM stattic, or 1 μM stattic for 24 h. **h** Western blot analysis of the indicated proteins in shSTAT3 A375 and SK-MEL-28 cells after the expression of the empty vector, wild-type STAT3 plasmid, or STAT3 phosphorylation mutant plasmids. **i** Viability of the indicated cells after treatment with sunitinib for 24 h. Two-tailed unpaired Student’s *t* test was performed in (**a**). Nonlinear regression was applied in (**e** and **g**). *P* values were calculated using one-way ANOVA in (**i**). ***P < 0.001.

STAT3 inhibition has been implicated in cell cycle arrest and apoptosis^26–28^. Additionally, the STAT3 signaling pathway is associated with drug sensitivity in melanoma^29–31^. Thus, we focused our attention on the association of STAT3 signaling with sunitinib sensitivity. By analyzing the associations between sunitinib sensitivity and the activity of STAT3 signaling using the Cancer Therapeutics Response Portal, we found that STAT3 signaling was significantly activated in cells with a higher IC50 of sunitinib (Fig. 3a). Consistent with these results, GSEA demonstrated that the IL6/JAK/STAT3 pathway was markedly activated in STAT3-resistant cells (Fig. 3b). We also generated sunitinib-resistant melanoma cells (Supplementary Fig. 2) and found that the phosphorylation of STAT3 was increased in sunitinib-resistant melanoma cells (Fig. 3c). Furthermore, we generated STAT3 knockdown melanoma cells by transduction of two different shRNAs (Fig. 3d; Supplementary Fig. 3a) and found that STAT3 inhibition significantly enhanced melanoma cell sensitivity to sunitinib (Fig. 3e). Stattic, an inhibitor of STAT3, which is reported to impede the phosphorylation of STAT3 (Fig. 3f), sensitized both A375 and SK-MEL-28 melanoma cells to sunitinib (Fig. 3g). STAT3 functions mainly through its phosphorylation on tyrosine (Y705) and serine (S727) residues, which is important for its dimerization, translocation and transcriptional activation^2^. Thus, we sought to determine the site of STAT3 phosphorylation that mediates melanoma cell sensitivity to sunitinib. In STAT3 knockdown cells, we ectopically expressed wild-type STAT3 or STAT3 phosphorylation mutants (Fig. 3h). Notably, coexpression of wild-type STAT3 but not the STAT3 phosphorylation mutants, attenuated the shSTAT3-induced sensitization of melanoma cells to sunitinib (Fig. 3i). Taken together, these results demonstrated that STAT3 inhibition enhances melanoma cell sensitivity to sunitinib via suppression of STAT3 phosphorylation.

### BET inhibitors suppress STAT3 signaling via the BRD4/IL6 axis

**Fig. 4:**
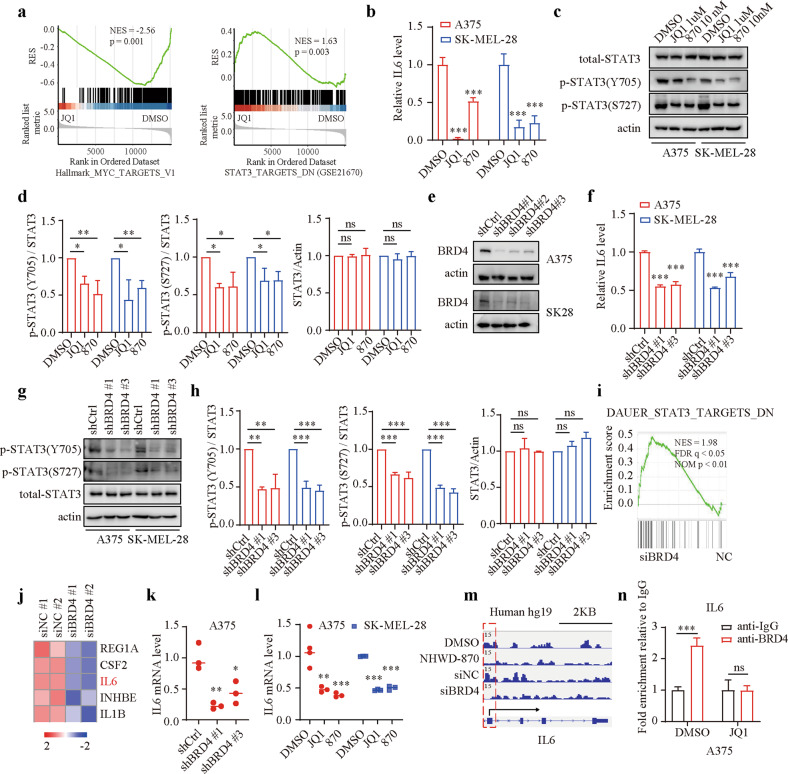
BET inhibitors repress STAT3 signaling via the BRD4/IL6 axis. **a** GSEA of MYC targets and targets with STAT3-induced downregulation in the JQ1 and DMSO groups. **b** IL6 concentrations in supernatants were measured by ELISA after treatment with DMSO, 1 μM JQ1, or 10 nM NHWD-870 for 24 h. **c**, **d** Western blotting and qualitative analysis of the indicated proteins in A375 and SK-MEL-28 cells after treatment with DMSO, 1 μM JQ1, or 10 nM NHWD-870 for 24 h. **e** Knockdown efficiency of BRD4 quantified by western blotting. **f** IL6 concentrations in supernatants were measured by ELISA after BRD4 silencing. **g**, **h** Western blotting and qualitative analysis of the indicated proteins in A375 and SK-MEL-28 cells after BRD4 silencing. **i** GSEA of targets with STAT3-induced downregulation identified by RNA-seq of siNC vs. siBRD4 A375 cells. **j** Heatmap of the top five differentially expressed genes in the gene set. **k** Assessment of the IL6 mRNA level by RT-PCR in A375 cells after BRD4 silencing. **l** Assessment of IL6 mRNA levels by RT-PCR in A375 and SK-MEL-28 cells after treatment with DMSO, 1 μM JQ1, or 10 nM NHWD-870 for 24 h. **m** BRD4 binding peaks in the IL6 promoter in DMSO-treated, NHWD-870-treated, siNC-treated, and siBRD4-treated A375 cells. **n** ChIP-qPCR analysis of the IL6 promoter in A375 cells with an anti-BRD4 antibody or IgG after treatment with DMSO or 1 μM JQ1. *P* values were calculated using one-way ANOVA in (**b**, **d**, **f**, **h**, **k** and **l**). Two-way ANOVA was performed in (**n**). *P < 0.05; **P < 0.01; ***P < 0.001; ns nonsignificant.

BET inhibitors have been reported to modulate drug sensitivity in cancer cells by specifically regulating the expression of certain genes or the activity of signaling pathways^11,12^. Thus, we sought to determine whether BET inhibitors can suppress STAT3 signaling to sensitize melanoma cells to sunitinib. We reanalyzed the RNA-seq data of JQ1-treated vs. control cells. As expected, the expression of c-Myc targets was dramatically inhibited after JQ1 treatment (Fig. 4a). However, the targets with STAT3-induced downregulation were significantly upregulated in the JQ1 group, suggesting that JQ1 can markedly inhibit IL6/STAT3 signaling activity (Fig. 4a). Subsequently, we measured the IL6 concentration in the supernatant using an ELISA kit and found that the IL6 concentration was markedly reduced in melanoma cells after BET inhibitor treatment (Fig. 4b). Consistent with these results, the phosphorylation of STAT3 was also dramatically decreased after BET inhibitor treatment (Fig. 4c, d).

BET inhibitors function primarily by competitively binding to BET proteins and disrupting the interaction between BET proteins and acetylated lysine residues^32^. To determine which BET proteins are responsible for IL6/STAT3 pathway signaling, we silenced BRD2/3/4 individually using shRNA (Fig. 4e; Supplementary Fig. 3b, c). The results showed that knockdown of only the BRD4 protein markedly reduced the IL6 concentration and the phosphorylation of STAT3 (Fig. 4f–h; Supplementary Fig. 3d). We also silenced BRD4 using siRNA and performed RNA-seq, finding that STAT3 signaling was markedly inhibited after BRD4 silencing (Fig. 4i). The top five differentially expressed genes in the gene set were visualized using a heatmap, which suggested that the mRNA level of IL6 was significantly reduced after BRD4 silencing (Fig. 4j). Consistent with the RNA-seq results, BRD4 knockdown and BET inhibitor treatment markedly reduced the mRNA level of IL6, according to quantitative real-time PCR analysis (Fig. 4k, l). Reanalysis of an existing BRD4 ChIP-seq dataset suggested that there is a striking BRD4 binding peak in the IL6 gene promoter, while the amplitude of the binding peak was diminished with NHWD-870 treatment or BRD4 knockdown (Fig. 4m). The results were also validated by ChIP-qPCR (Fig. 4n; Supplementary Fig. 3e). These findings suggested that BET inhibitors suppress STAT3 signaling via the BRD4/IL6 axis.

### BET inhibitors regulate sunitinib sensitivity by inhibiting STAT3 activity and GDF15 expression

**Fig. 5:**
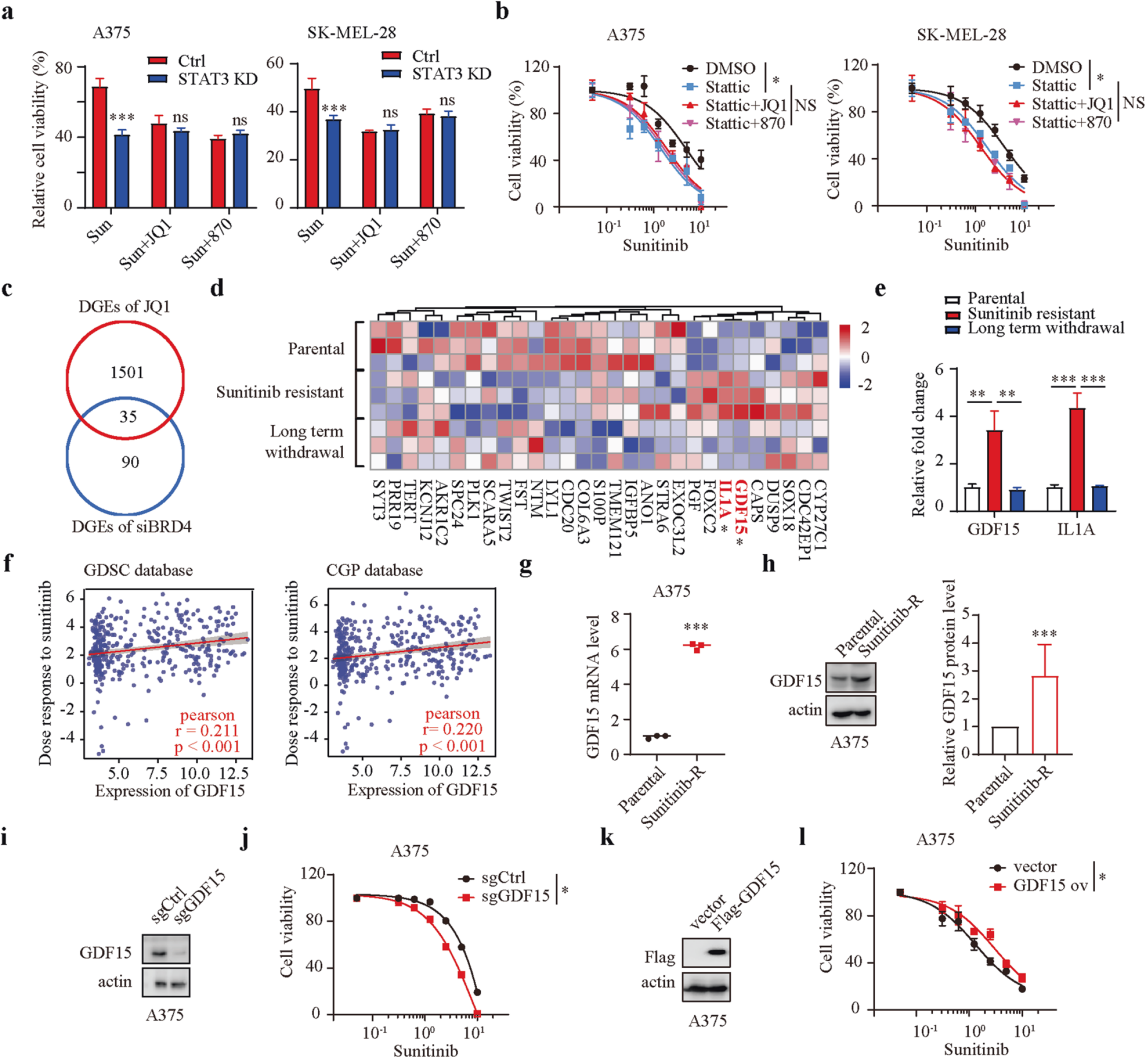
BET inhibitors regulate sunitinib sensitivity by inhibiting STAT3 activity and GDF15 expression. **a** The viability of shCtrl and shSTAT3 melanoma cells was evaluated after treatment with sunitinib (1 μM) alone or in combination with JQ1 (1 μM)/NHWD-870 (10 nM). **b** Dose response of sunitinib-induced death in A375 and SK-MEL-28 cells in the presence of DMSO, 1 μM stattic, or 1 μM stattic + JQ1 (1 μM)/NHWD-870 (10 nM). **c** Venn diagram of the overlapping genes in the indicated groups. **d** Heatmap of the overlapping genes in parental cells, sunitinib-resistant cells, and cells with long-term withdrawal of sunitinib from the GSE122821 dataset. If the mRNA level of a gene was markedly increased in sunitinib-resistant cells and comparable to that in parental cells after long-term withdrawal of sunitinib, the gene is marked with an asterisk and shown in red. **e** Fold changes in GDF15 and IL1A expression in parental cells, sunitinib-resistant cells, and cells with long-term withdrawal of sunitinib from the GSE122821 dataset. **f** Association between the expression of GDF15 and the logIC50 of sunitinib in Genomics of Drug Sensitivity in Cancer and Cancer Genome Project datasets. **g** GDF15 mRNA levels in parental and sunitinib-resistant A375 cells. **h** Western blotting and qualitative analysis of GDF15 expression in parental and sunitinib-resistant A375 cells. **i** GDF15 protein levels were quantified by western blotting in control (sgCtrl) and GDF15-deficient (sgGDF15) cells. **j** Dose response of sunitinib-induced death in sgCtrl and sgGDF15 A375 cells over a 24 h period. **k** GDF15 protein levels were quantified by western blotting in control (Flag vector) and GDF15 overexpression (Flag-GDF15) cells. **l** Dose response of sunitinib-induced death in vector and GDF15-overexpressing A375 cells over a 24 h period. Two-way ANOVA was performed in (**a**). Nonlinear regression was applied in (**b**, **j** and **l**). One-way ANOVA was performed in (**e**). Two-tailed unpaired Student’s *t* test was performed in (**g** and **h**). *P < 0.05; **P < 0.01; ***P < 0.001.

We next sought to determine whether STAT3 signaling mediates BET inhibitor-induced sensitization of melanoma cells to sunitinib. Control and STAT3-silenced melanoma cells were incubated with sunitinib or cotreated with sunitinib and BET inhibitors. A marked degree of sensitization to sunitinib was observed after BET inhibitor treatment in control cells but not in STAT3-silenced cells (Fig. 5a). Pharmacologically, stattic sensitized melanoma cells to sunitinib but failed to further enhance the sensitization of melanoma cells to sunitinib in the presence of BET inhibitors (Fig. 5b). These results suggested that STAT3 signaling is the mediator of BET inhibitor-induced sensitization of melanoma cells to sunitinib. To further elucidate the specific protein that mediates BET inhibitor-induced sensitization of melanoma cells to sunitinib, we overlapped the downregulated differentially expressed genes after BET inhibitor treatment and after BRD4 silencing (Fig. 5c). A Venn diagram identified 35 DEGs, among which GDF15 and IL1A exhibited markedly increased mRNA levels in sunitinib-resistant cells and comparable mRNA levels after long-term withdrawal of sunitinib (Fig. 5d, e). We further found that the expression of GDF15 but not IL1A was positively associated with the logIC50 of sunitinib using Genomics of Drug Sensitivity in Cancer (GDSC) and Cancer Genome Project (CGP) datasets (Fig. 5f; Supplementary Fig. 4a, b). Subsequently, quantitative real-time PCR analysis validated that the mRNA level of GDF15 was markedly reduced after BET inhibitor treatment (Supplementary Fig. 4c). GDF15 expression was significantly increased in sunitinib-resistant melanoma cells (Fig. 5g, h; Supplementary Fig. 4d, e). We further generated GDF15 knockout cells using CRISPR/Cas9 technology (Fig. 5i; Supplementary Fig. 4f) and found that GDF15 knockout sensitized melanoma cells to sunitinib (Fig. 5j; Supplementary Fig. 4g). In contrast, GDF15 overexpression reduced cell sensitivity to sunitinib (Fig. 5k, l; Supplementary Fig. 4h, i). These findings suggested that BET inhibitors regulate melanoma cell sensitivity to sunitinib by inhibiting STAT3 activity and GDF15 expression.

### BRD4 regulates GDF15 expression by directly targeting its promoter or indirectly targeting the IL6/STAT3 axis

**Fig. 6:**
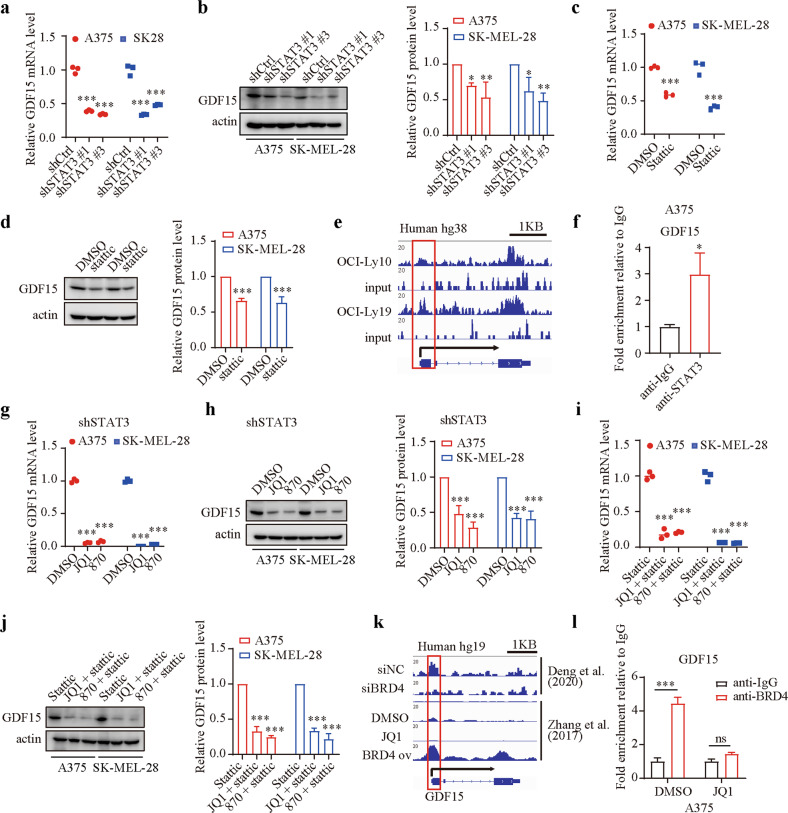
BRD4 regulates GDF15 expression by directly targeting its promoter or indirectly targeting the IL6/STAT3 axis. **a** GDF15 mRNA levels in A375 and SK-MEL-28 cells after STAT3 silencing. **b** Western blotting and qualitative analysis of GDF15 expression in control and STAT3-silenced cells. **c** GDF15 mRNA levels in A375 and SK-MEL-28 cells after stattic treatment. **d** Western blotting and qualitative analysis of GDF15 expression in DMSO- and stattic-treated cells. **e** STAT3 binding peak in the GDF15 promoter in OCI-Ly10 and OCI-Ly19 cells (GSE50723). **f** Validation of STAT3 binding to the promoter of GDF15 in A375 cells by ChIP-qPCR. **g** GDF15 mRNA levels in STAT3-silenced cells after treatment with DMSO, 1 μM JQ1, or 10 nM NHWD-870 for 24 h. **h** Western blotting and qualitative analysis of GDF15 expression in STAT3-silenced cells after treatment with DMSO, 1 μM JQ1, or 10 nM NHWD-870 for 24 h. **i** GDF15 mRNA levels in stattic-treated cells after treatment with DMSO, 1 μM JQ1, or 10 nM NHWD-870 for 24 h. **j** Western blotting and qualitative analysis of GDF15 expression in stattic-treated cells after treatment with DMSO, 1 μM JQ1, or 10 nM NHWD-870 for 24 h. **k** BRD4 binding peak in the GDF15 promoter in siNC-treated and siBRD4-treated A375 cells (upper); BRD4 binding peak in the GDF15 promoter in DMSO-treated, JQ1-treated and BRD4-overexpressing cells (lower). **l** ChIP-qPCR analysis of the GDF15 promoter in A375 cells with an anti-BRD4 antibody or IgG after treatment with DMSO or 1 μM JQ1. One-way ANOVA was performed in (**a**, **b**, **g**, **h**, **i** and **j**). Two-tailed unpaired Student’s *t* test was performed in (**c** and **d**). *T* test with Welch’s correction was performed in (**f**). Two-way ANOVA was performed in (**l**). *P < 0.05; **P < 0.01; ***P < 0.001; ns nonsignificant.

To further elucidate the regulatory mechanism of GDF15 in melanoma cells, we first measured the expression of GDF15 in STAT3-silenced cells and found that the expression of GDF15 in STAT3-silenced cells was significantly reduced compared with that in control cells (Fig. 6a, b). The expression of GDF15 was also dramatically decreased after stattic treatment (Fig. 6c, d). Analysis of published ChIP-seq data demonstrated that there was a strikingly enhanced STAT3‐binding peak in the GDF15 promoter in both OCI-Ly10 and OCI-Ly19 cells (Fig. 6e). Based on the identified peak, we designed primers for ChIP‐qPCR analysis, confirming that STAT3 binds to the GDF15 promoter in melanoma cells (Fig. 6f; Supplementary Fig. 4j). It is worth noting that BET inhibitor treatment inhibited GDF15 expression more strongly than STAT3 silencing or stattic treatment (Supplementary Fig. 4c, k), suggesting that BET inhibitors suppress GDF15 expression in another manner in addition to through the BRD4/IL6/STAT3 axis. We previously showed that BRD4 could function as a transcription factor to regulate the expression of certain genes^10–12^. Therefore, we sought to determine whether BRD4 can directly regulate the expression of GDF15. We incubated STAT3-silenced cells with BET inhibitors and found that the expression of GDF15 was significantly reduced (Fig. 6g, h), which is in line with the findings in stattic-treated cells (Fig. 6i, j). Reanalysis of ChIP-seq data suggested that BRD4 binding to the GDF15 promoter was diminished by BRD4 inhibition and enhanced in BRD4-overexpressing cells (Fig. 6k). These findings were validated by ChIP-qPCR (Fig. 6l; Supplementary Fig. 4l). To further visualize the binding sites of BRD4 and STAT3 in the GDF15 promoter, the ENCODE database and UCSC Genome Browser were used and showed that there were strikingly enhanced peaks in the GDF15 promoter and that the peaks of BRD4 and STAT3 in the GDF15 promoter did not overlap (Supplementary Fig. 5). These results indicated that GDF15 is transcriptionally regulated directly by BRD4 or indirectly by the BRD4/IL6/STAT3 axis.

### Combination of BET inhibitors with sunitinib causes melanoma suppression in vivo

**Fig. 7:**
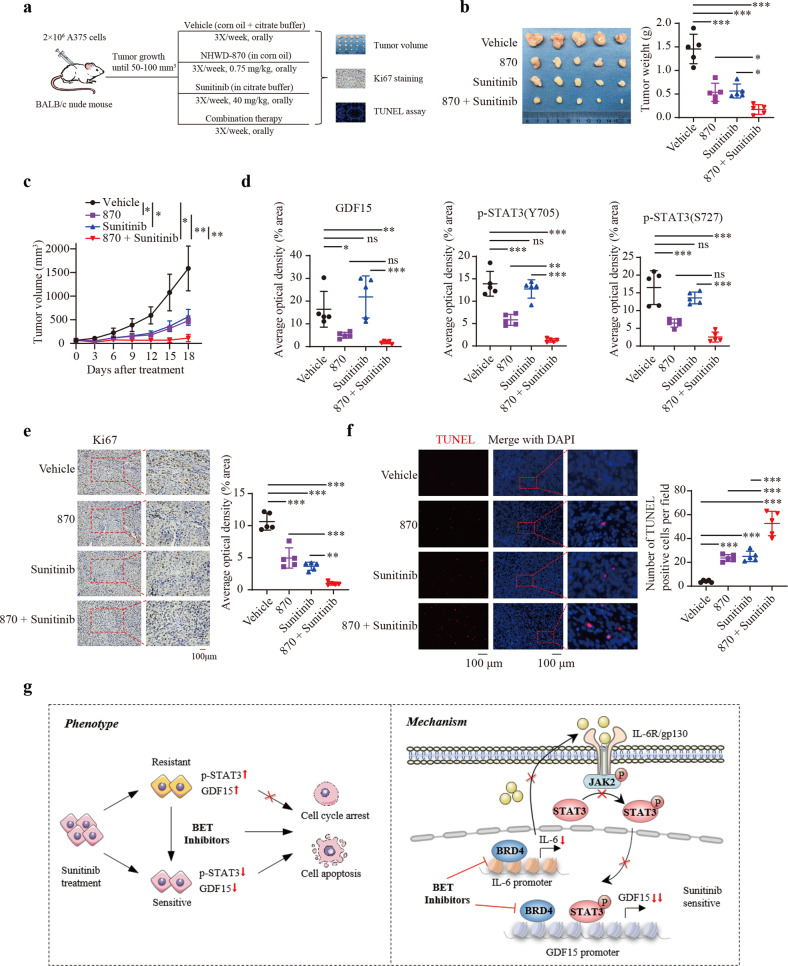
Combination treatment with BET inhibitors and sunitinib causes melanoma suppression in vivo. **a** Schedule for administration of sunitinib (40 mg/kg) and NHWD-870 (0.75 mg/kg) in tumor-bearing mice. **b**, **c** Tumor weight (**b**) and tumor volume (**c**) in the vehicle, NHWD-870, sunitinib, and combination groups. **d** Quantification of IHC staining of GDF15, p-STAT3 (Y705), and p-STAT3 (S727) in the sectioned tumors. **e** Ki67 staining of the sectioned tumors was performed to identify tumor cell proliferation in the vehicle, sunitinib, NHWD-870 and combination groups. **f** A TUNEL assay was performed to quantify apoptotic cells in xenograft tumors in the vehicle, sunitinib, NHWD-870 and combination groups. **g** A proposed working model. BET inhibitors synergize with sunitinib in melanoma cells by further inducing apoptosis and cell cycle arrest. Mechanistically, BET inhibitors sensitize melanoma cells to sunitinib by inhibiting the IL6/STAT3 signaling pathway and GDF15 expression. GDF15 is transcriptionally directly regulated by BRD4 or indirectly regulated by the BRD4/IL6/STAT3 axis. One-way ANOVA was performed in (**b**, **d**, **e** and **f**). Brown-Forsythe and Welch ANOVA was performed in (**c**). *P < 0.05; **P < 0.01; ***P < 0.001; ns nonsignificant.

To evaluate the therapeutic potential of combining BET inhibitors with sunitinib in melanoma, A375 cells were inoculated into the right flanks of nude mice to establish subcutaneous xenograft models. When the tumor volume reached 50-100 mm^3^, the tumor-bearing mice were randomly allocated into groups as follows: vehicle, sunitinib, NHWD-870 or combination treatment using sunitinib and NHWD-870. All treatments were administered for two successive days and stopped for 1 day (Fig. 7a). The results showed that the tumor volume and weight were further markedly decreased in the combination therapy group compared with the other groups, without significant changes in body weight (Fig. 7b, c; Supplementary Fig. 6a). Real-time PCR analysis of tumors showed that GDF15 and IL6 mRNA expression was significantly decreased in the NHWD-870 and combination therapy groups (Supplementary Fig. 6b, c). IHC staining also showed that the protein levels of GDF15, p-STAT3 (Y705) and p-STAT3 (S727) were significantly decreased in the NHWD-870 and combination therapy groups (Fig. 7d; Supplementary Fig. 6d). IHC staining for the proliferation marker Ki67 revealed fewer proliferative cells in the combination therapy group (Fig. 7e). The TUNEL assay indicated a significantly increased number of apoptotic cells in the combination therapy group (Fig. 7f). These results further supported the synergistic effect of the combination of BET inhibitors and sunitinib on inducing tumor suppression in vivo. Taken together, our results strongly demonstrated that combination therapy using BET inhibitors and sunitinib has immense therapeutic potential in melanoma. Mechanistically, BET inhibitors sensitize melanoma cells to sunitinib by inhibiting the BRD4/GDF15 axis and the BRD4/IL6/STAT3/GDF15 axis (Fig. 7g).

## Discussion

BET inhibitors, including NHWD-870 developed by our team, have shown profound efficacy against hematologic and solid tumors in preclinical studies^9,10,33^. However, several resistance mechanisms limit the efficacy of BET inhibitors in melanoma. For example, Ambrosini et al. demonstrated that NF-κB signaling was significantly activated in BETi-resistant melanoma cells and that inhibition of NF-κB signaling enhanced BET inhibitor sensitivity in melanoma^34^. Moreover, Chua et al. showed that FGF2 secreted from hepatic stellate cells (HSCs) protected melanoma cells against growth inhibition induced by BET inhibitors, identifying a potential mechanism underlying the resistance of melanoma liver metastases to BET inhibitors^35^. Therefore, it is of great significance to develop novel strategies to better kill melanoma cells with BET inhibitor treatment.

In the present study, we identified sunitinib as a clinically applicable drug that synergizes with BET inhibitors in melanoma cells through a screen of 240 FDA-approved antitumor drugs. Sunitinib is an indolinone derivative and tyrosine kinase inhibitor approved for clear cell renal cell carcinoma and gastrointestinal stromal tumor treatment. We previously demonstrated that sunitinib had a therapeutic effect on melanoma^21^. Moreover, Minor et al. showed that melanoma patients with KIT mutations had a good response to sunitinib^36^. Sunitinib is well known to exert antiangiogenic activity by inhibiting CSF1R, CSF3R, FLT1, FLT3, FLT4, KDR, KIT, PDGFRA, PDGFRB and RET. By analyzing the associations of these targets with immunosuppressive cells and inhibitory immune checkpoints, we previously found that sunitinib treatment was associated with T-cell infiltration and activity and that sunitinib showed a synergistic antitumor effect with an anti-CTLA-4 monoclonal antibody in melanoma through the P62/PD-L1 axis^20^. Additionally, sunitinib was reported to play a critical role in immune surveillance by inducing a Th-1 immune response or reducing the population of MDSCs^37,38^. Here, we further revealed that BET inhibitors synergize with sunitinib in melanoma. These findings highlight the importance of sunitinib in melanoma treatment.

We further showed that BET inhibitors sensitize melanoma cells to sunitinib by repressing GDF15 expression in a direct way by targeting its promoter or indirectly by targeting the IL6/STAT3 axis. GDF15, also called NSAID-activated gene-1 (NAG-1), is associated with multiple biological processes and diseases, including cancer^39^. GDF15 expression was found to be increased in melanoma metastases compared with benign nevi or primary melanomas and was positively correlated with stage in melanoma patients^40^. High GDF15 serum levels were also correlated with poorer overall survival in melanoma patients^41^. In addition, GDF15 has been extensively reported to act as an antiapoptotic protein^42–46^. Mechanistically, GDF15 inhibits apoptosis by promoting rapid and transient Ser473 phosphorylation (activation) of Akt and a subsequent increase in Ser136 phosphorylation (inactivation) of Bad, which is well known to promote apoptosis through the intrinsic mitochondrial pathway^43^. This finding was consistent with Liu et al.’s study showing that inhibition of GDF15 stabilized PTEN, in turn inactivating the PI3K/AKT pathway and finally inducing cancer cell apoptosis^44^. As expected, increased expression of GDF15 in B16F1 melanoma cells promoted tumor growth in the B16F1 melanoma mouse model^47^. Therefore, GDF15 might be a potential therapeutic target in melanoma.

The association between GDF15 expression and sunitinib sensitivity has never been reported. Sunitinib was reported to induce cell apoptosis in renal cell carcinoma via STAT3 inhibition^48^. We further demonstrated that STAT3 signaling was significantly activated in sunitinib-resistant melanoma cells and that inhibition of STAT3 enhanced the sensitivity of melanoma to sunitinib. Furthermore, inhibition of STAT3 decreased the expression of GDF15, and GDF15 silencing increased the sensitivity of melanoma cells to sunitinib. These results implied that there is a negative association between GDF15 expression and sunitinib sensitivity. Considering the antiapoptotic role of GDF15, it is reasonable to speculate that GDP15 leads to sunitinib resistance in melanoma by inhibiting apoptosis.

Additionally, BET inhibitors can directly regulate GDF15 expression by competitively binding to BRD4 and reducing its distribution in the promoter of GDF15. These results were validated in STAT3 knockdown and stattic-treated cells. The direct regulation of GDF15 expression by BRD4 has never been clarified. Guo et al. recently showed that BRD4 upregulated the expression of GDF15 by inducing NR5A2 transcriptional activation^49^. However, this group failed to evaluate the direct association of BRD4 and GDF15. Through ChIP‒qPCR, we found that BRD4 directly bound to the promoter of GDF15 and that the binding was decreased by BRD4 silencing or inhibition and promoted by BRD4 overexpression. Consistent with these results, the xenograft assays further validated that GDF15 expression was significantly decreased in the BET inhibitor-treated group and combination therapy group compared to the DMSO and sunitinib groups.

In summary, we identified an FDA-approved drug, sunitinib, that synergizes with BET inhibitors in melanoma. Mechanistically, BET inhibitors sensitize melanoma cells to sunitinib by inhibiting the BRD4/GDF15 axis and BRD4/IL6/STAT3/GDF15 axis. These findings are potentially translatable toward novel therapies for melanoma and other diseases that can be cotreated with BET inhibitors and sunitinib.

## Supplementary information

Supplementary figures
