# Supplementary material for: BET inhibitors synergize with sunitinib in melanoma through GDF15 suppression
Source: Exp Mol Med. 2023 Feb 1;55(2):364–76. doi: 10.1038/s12276-023-00936-y (PMC9981764; doi:10.1038/s12276-023-00936-y)
Supplement: Supplementary file 1 — Supplementary figures [file 12276_2023_936_MOESM1_ESM.pdf]

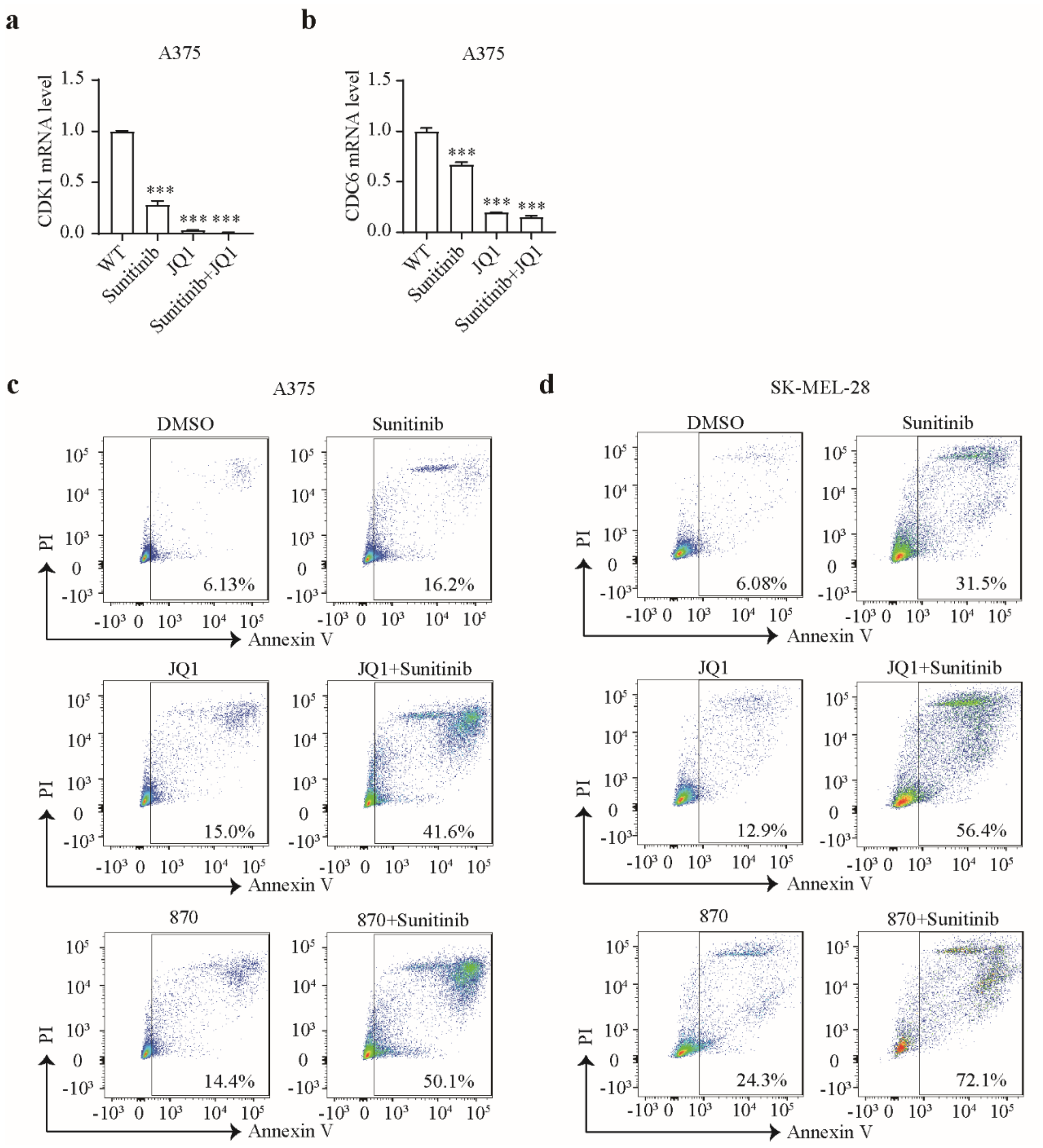

Supplementary Fig. 1. Sunitinib synergize with BET inhibitors in melanoma. (**a-b**). Assessment of CDK1 (**a**) and CDC6 (**b**) by RT-PCR in A375 cells after treatment with DMSO, sunitinib (1 µM), JQ1 (1 µM), or a combination of both drugs. (**c-d**) Apoptosis of A375 (**c**) and SK-MEL-28 (**d**) cells after treatment with sunitinib (1 µM) or JQ1 (1 µM) / NHWD-870 (10 nM) either alone or in combination for 36 h. P values were calculated using one-way ANOVA analysis. ***, $P < 0.001$.

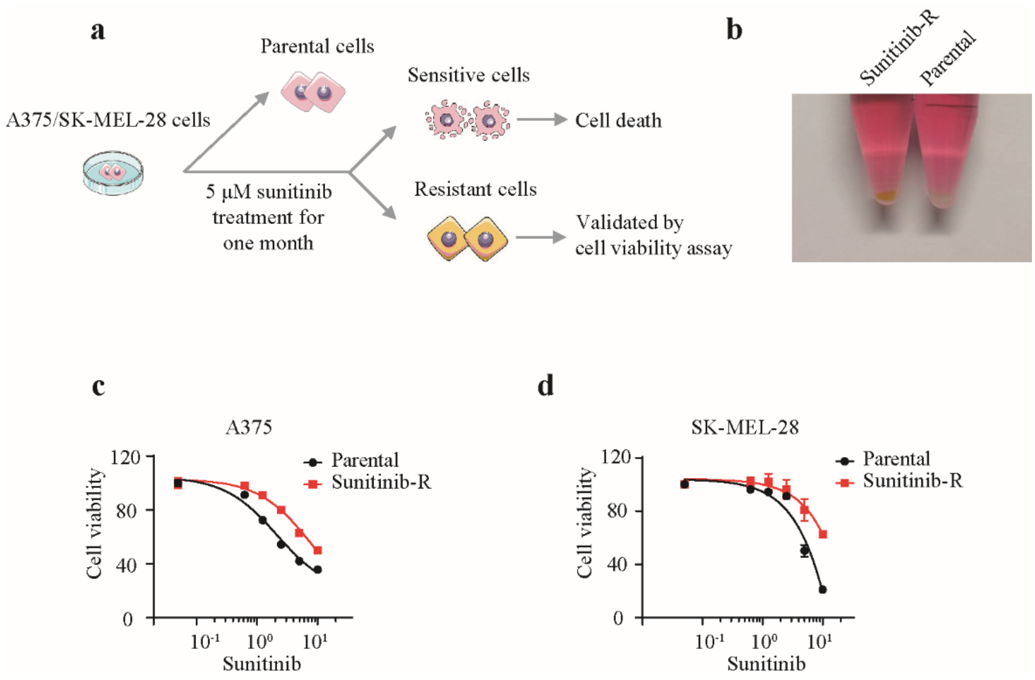

Supplementary Fig. 2. Construction of sunitinib resistant cells. (**a**) Scheme used to generate sunitinib resistant cells. (**b**) Picture of parental and sunitinib resistant cells. (**c-d**) Dose response of sunitinib-induced death of parental and sunitinib resistant cells.

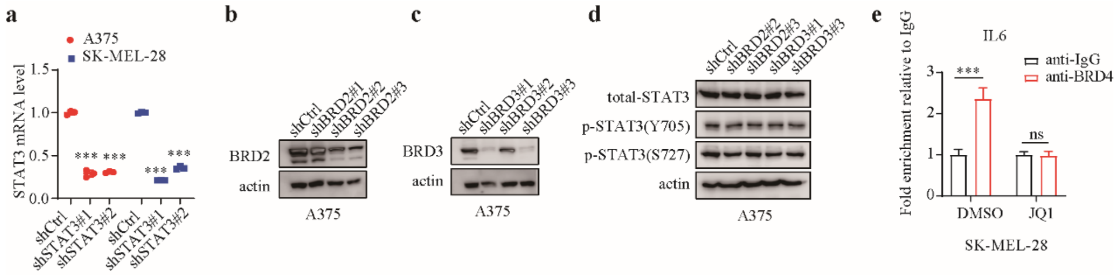

Supplementary Fig. 3. BET inhibitors repress STAT3 signaling via BRD4/IL6 axis. (**a**) Assessment of STAT3 by RT-PCR in A375 and SK-MEL-28 cells after STAT3 silencing. (**b**) Western blotting analysis of BRD2 in A375 cells after BRD2 silencing. (**c**) Western blotting analysis of BRD3 in A375 cells after BRD3 silencing. (**d**) Western blotting analysis of the indicated proteins in A375 cells after BRD2 or BRD3 silencing. (**e**) ChIP-qPCR analysis of the IL6 promoter in SK-MEL-28 cells with BRD4 or IgG antibody after treatment with DMSO or 1 μM JQ1. P values were calculated using one-way ANOVA analysis in **a**. Two-way ANOVA analysis was performed in **e**.***, P < 0.001; ns, no significance.

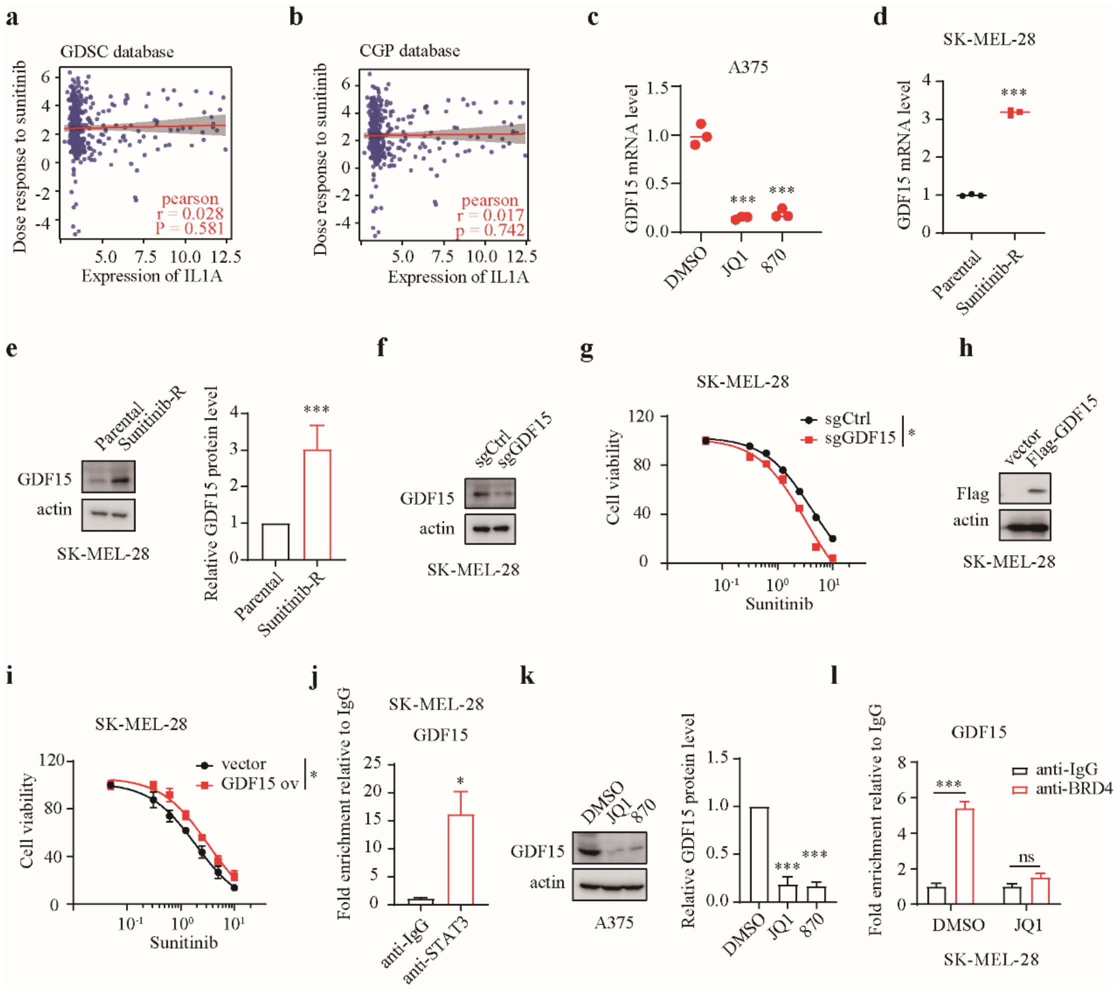

Supplementary Fig. 4. BET inhibitors regulate sunitinib sensitivity through inhibiting GDF15 expression. (**a-b**) Association between expression of IL1A and logIC50 of sunitinib in Genomics of Drug Sensitivity in Cancer (**a**) and Cancer Genome Project datasets (**b**). (**c**) Real-time PCR analysis of GDF15 expression in A375 cells after treatment with DMSO, 1 μM JQ1, or 10 nM NHWD-870 for 24 h. (**d**) GDF15 mRNA level in parental and sunitinib resistant SK-MEL-28 cells. (**e**) Western blotting and qualitative analysis of GDF15 expression in parental and sunitinib resistant SK-MEL-28 cells. (**f**) GDF15 protein levels were quantified by western blotting in control (sgCtrl) and GDF15 deficient (sgGDF15) cells. (**g**) Dose response of sunitinib-induced death of sgCtrl and sgGDF15 SK-MEL-28 cells for 24 h. (**h**) GDF15 protein levels were quantified by western blotting in control (Flag vector) and GDF15 overexpression (Flag-GDF15) cells. (**i**) Dose response of sunitinib-induced death of vector and GDF15 overexpression SK-MEL-28 cells for 24 h. (**j**) Validation of STAT3 binding to the promoter of GDF15 in SK-MEL-28 cells by ChIP-qPCR. (**k**) Western blotting analysis of GDF15 in A375 cells after JQ1 or NHWD-870 treatment. (**l**) ChIP-qPCR analysis of the GDF15 promoter in SK-MEL-28 cells with BRD4 or IgG antibody after treatment with DMSO or 1 μM JQ1. One-way ANOVA analysis was performed in **c** and **k**. Nonlinear regression was applied in **g** and **i**. Two-tailed unpaired Student's t-test was performed in **d**, **e**, and **j**. Two-way ANOVA analysis was performed in **l**. *, $P < 0.05$; ***, $P < 0.001$; ns, no significance.

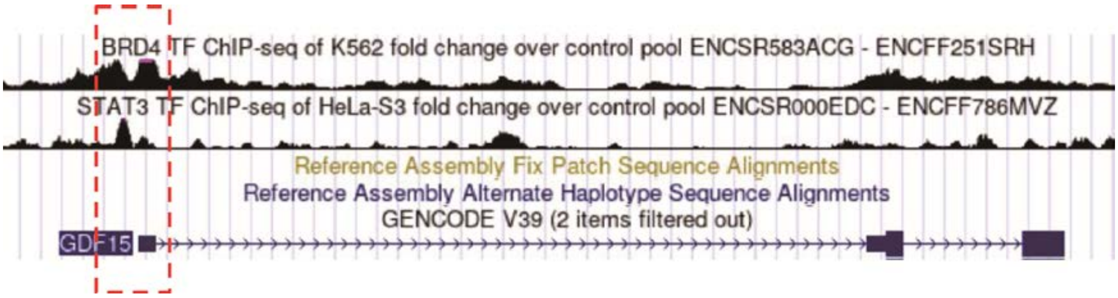

Supplementary Fig. 5. BRD4 binding peak in GDF15 promoter among K562 cells in GSE101225 (upper) and STAT3 binding peak in GDF15 promoter among HeLa-S3 cells in GSE31477 (lower).

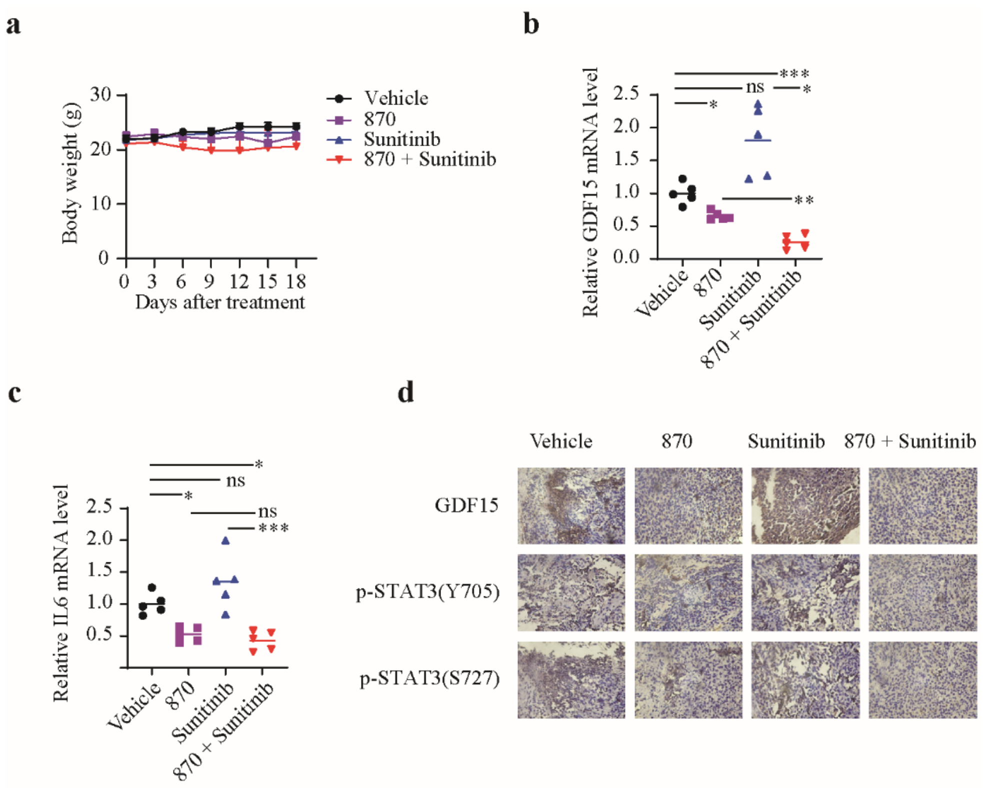

Supplementary Fig. 6. Combination of BET inhibitors with sunitinib causes melanoma repression in vivo. (**a**) Body weight in vehicle, NHWD-870, sunitinib, and combination groups. (**b**) GDF15 mRNA level quantified by real-time PCR in xenografted tumors. (**c**) IL6 mRNA level quantified by real-time PCR in xenografted tumors. (**d**) IHC staining of GDF15, p-STAT3(Y705), and p-STAT3(S727) in the sectioned tumors. P values were calculated using one-way ANOVA analysis. *, $P < 0.05$; **, $P < 0.01$; ***, $P < 0.001$; ns, no significance.
